# *In Silico* Investigation of Conformational Motions in Superfamily 2 Helicase Proteins

**DOI:** 10.1371/journal.pone.0021809

**Published:** 2011-07-19

**Authors:** Holger Flechsig, Denny Popp, Alexander S. Mikhailov

**Affiliations:** 1 Department of Physical Chemistry, Fritz Haber Institute of the Max Planck Society, Berlin, Germany; 2 Department of Physical Chemistry, Technical University Bergakademie Freiberg, Freiberg, Germany; German Cancer Research Center, Germany

## Abstract

Helicases are motor proteins that play a central role in the metabolism of DNA and RNA in biological cells. Using the energy of ATP molecules, they are able to translocate along the nucleic acids and unwind their duplex structure. They have been extensively characterized in the past and grouped into superfamilies based on structural similarities and sequential motifs. However, their functional aspects and the mechanism of their operation are not yet well understood. Here, we consider three helicases from the major superfamily 2 - Hef, Hel308 and XPD - and study their conformational dynamics by using coarse-grained relaxational elastic network models. Specifically, their responses to mechanical perturbations are analyzed. This enables us to identify robust and ordered conformational motions which may underlie the functional activity of these proteins. As we show, such motions are well-organized and have large amplitudes. Their possible roles in the processing of nucleic substrate are discussed.

## Introduction

Motor proteins are complex macromolecules, perfected by biological evolution and largely contributing to the activity of biological cells [1]. They are nanoscale engines that, powered by chemical energy derived from ATP molecules, perform mechanical motions which carry out a large variety of functions including transport of organelles along microtubules, contraction of muscles, transmembrane exchange of ions or control of flagellar motion.

An important class of motor proteins operating on nucleic acids are helicases [2], [3]. They are involved in DNA and RNA manipulations, such as the displacement of bound proteins, the removal of secondary structures and, most prominently, the separation of their duplex structures into single-stranded forms [4], [5]. Therefore, they play a major role in replication, recombination and repair of nucleic acid substrates. In viruses, helicases are an essential part of the molecular replication machinery and thus represent one of the main targets in antiviral therapies [6], [7]. The energy needed to carry out this variety of processes is harnessed from the enzymatic activity of the helicases, i.e. their ability to catalyze the conversion of ATP molecules into ADP and a phosphate upon binding them [8]–[10]. It is known that helicase proteins can undergo substantial conformational changes accompanying their operation [8]–[13].

In the past, helicases from different organisms have been identified, characterized and grouped into superfamilies based on sequence comparisons (see [14] and the more recent review [8]). Today, the crystal structure of many helicases has been resolved - for some of them also in a complexed form with bound ligands. Recently, crystallographic snapshots of helicase-complexes liganded with ATP-analogs, ADP and the nucleic acid have been determined, yielding valuable insights into their functional modes [11]–[13]. Furthermore, time-resolved single-molecule experiments have been performed for the prototype helicase of the hepatitis C virus [15], [16].

Despite the fact that the number of different helicases adopted by organisms to perform manipulation of DNA and/or RNA molecules is large, it has turned out that many of them have a similar fold. Among helicases from the major superfamily 2, this similarity is reflected in the presence of two structurally related neighboring core domains [8]. Additional structurally diverse domains contribute to the overall architecture of the helicases. Besides of structural aspects, proteins in this superfamily also share conserved residue motifs that are located in different parts of the proteins and associated with specific functions related to ATP ligands and the nucleic acid [8], [17]. Important motifs involved in binding of ATP are found in the core domains, among them the well-known Walker A motif (also referred to as motif I), the Walker B motif (referred to as motif II) and motif VI.

For the ATP-dependent motor activity of superfamily 2 helicases, the following mechanism could be deduced from the structural data and comparison with functionally similar proteins [2], [8]–[10], [18]: A motor cycle is activated by binding of an ATP molecule inside the cleft between the core motor domains making contacts to the conserved motifs located there. This leads to conformational changes and relative motions of the core motor domains with respect to each other are induced, so that the hydrolysis of ATP becomes possible. The hydrolysis reaction, in turn, produces substantial conformational motions. The ejection of chemical product completes the cycle and resets the initial conformation.

Relative motions of the core motor domains in superfamily 2 helicases can have a large amplitude. Under binding of ATP, these proteins can undergo a change from the open conformation, where the motor domains are separated, to a closed conformation with the motor domains being adjacent [11]–[13]. Combined with a gripping of the two motor domains to the nucleic acid strand, such opening and closing motions can account for translocation along the strand. Subsequent cycles, initiated by binding of an ATP molecule, can produce steady locomotion of the helicase along the nucleic acid. For the best studied member of superfamily 2, the NS3 helicase of hepatitis C virus (HCV), such an inchworm ratchet mechanism has been previously proposed based on the structural data [19] and the results of single-molecule experiments [16].

Investigations of helicase functioning usually rely on static structural information. The experiments do not yet allow to follow dynamical conformational changes in proteins within their functional cycles. Therefore, theoretical modeling can play an important role here, helping to understand the operation principles of helicases as molecular motors. To do this, the models should be able to describe functional conformational motions.

The most accurate descriptions of protein dynamics are provided by all-atom molecular dynamics (MD) simulations. Due to high computational expense, their applications are, however, restricted to molecular processes that occur on short timescales, often spatially localized in some regions of the protein, such as binding of a ligand or dissociation of chemical products. Modeling of entire turnover cycles in motor proteins, with the timescales typically ranging from tens of milliseconds to seconds, lies beyond the feasibility of all-atom MD simulations on current computer architectures.

Therefore, coarse-grained models of reduced complexity are needed to investigate functional conformational dynamics of protein motors. Such models can be obtained by applying approximations at the structural level as well as concerning the interactions inside the protein (see [20] and the more recent review [21]).

One popular approach is provided by elastic network (EN) models which treat a protein as a deformable elastic object [22]–[24]. In this case, structural coarse-graining is typically carried out by replacing amino acid residues by single point particles and by approximating interactions between them through empirical harmonic potentials. Using crystallographic data, the protein is thus mechanically modeled as a network of identical beads connected by elastic springs. Since the pioneering work by Tirion [22], such reduced descriptions have been applied to a variety of proteins [25]. Despite their simplicity, such models are able to remarkably well predict ligand-induced conformational changes [24]. EN studies of helicase proteins have been previously undertaken [26]. It should be noted that many EN studies are based on the computation of normal modes of elastic networks. However, EN models beyond the harmonic approximation have also been applied to study relaxational motions in motor proteins [27] and nonlinearities in mechanochemical motions of myosin and kinesin motors have been discussed [28].

Recently, we were able to trace entire operation cycles of the HCV NS3 helicase in a structurally resolved manner by using an EN model for this protein and including its interactions with DNA and ATP [29]. We have identified large-scale ordered motions inside the protein that bring together the two motor domains or spatially separate them depending on the presence of the ATP ligand. The switching between open and closed protein conformations can drive translocation and, as we have shown, HCV helicase can move along the nucleic acid chain by one base per cycle induced by binding of one ATP molecule. It has been furthermore demonstrated that the third domain of HCV helicase acts as a wedge which is dragged between the two nucleic acid strands and mechanically separates them.

While significant experimental and theoretical progress in understanding the function of HCV helicase has been made, operation mechanisms of other helicases in the same superfamily, especially those with limited structural data available, remain less clear. The open questions particularly refer to the ATP-dependent conformational motions inside these proteins and their coupling to the activity on the nucleic acid. Whether the *one step translocation*, i.e. the base-by-base motion of the motor domains along the nucleic acid consuming one ATP in each cycle, represents the common mode of helicase locomotion is a topic of current debate [9], [18]. The mechanistic role of other structural domains in the processing of duplex DNA/RNA substrates by other helicases from superfamily 2 is also not certain.

Our present study is focused on the analysis of large-amplitude conformational motions in the superfamily 2 helicases. It has been performed within the EN approximation, previously used to consider functional motions in HCV helicase [29]. Moreover, the same methods as in Ref. [29] have been used here to analyze mechanical responses of the selected proteins. This allows us to compare the properties of conformational motions for the chosen helicases with those of the previously investigated HCV helicase.

Our investigations have been performed for the Hef helicase from *Pyrococcus furiosus* that manipulates fork-structured DNA forms [30], the Hel308 helicase from *Archaeoglobus fulgidus* involved in unwinding of lagging strands on replication forks and other branched nucleic acids [31], and the XPD helicase from *Sulfolobus tokodaii* that opens DNA duplex structures during transcription and repair processes [32]. We construct the elastic networks for the proteins and investigate their dynamical behavior in response to various kinds of deformations.

Our primary aim is to find out whether the considered helicases, similar to other previously studied motor proteins [27]–[29], possess robust and well-defined conformational motions; we also want to discuss the possible functional role of such motions in different helicases. Therefore, we shall generate a large number of random initial deformations and consider conformational relaxation processes starting from them. In this way, robust conformational motions can be identified for each of the proteins. In the actual operation cycles of these molecular machines, functional conformational motions are induced by the perturbations localized near the nucleotide binding site and involving binding of ATP, the hydrolysis reaction and release of the products. The coarse-grained nature of the elastic-network models does not allow us to include specific chemical details of all such processes. Nonetheless, we can still analyze the related conformational responses by applying various random perturbations (forces) to the residues which belong to the experimentally known conserved motifs and are expected to be involved in the ATP binding and hydrolysis reaction. Once robust ordered conformational motions are identified by following changes in the distances between three selected labels, we shall look more carefully at such motions, producing a sequence of conformational snapshots and generating a video of the conformational motion. Finally, possible functional roles of identified motions will be discussed, with a special emphasis on DNA processing by the considered helicases.

## Results

**Figure 1:**
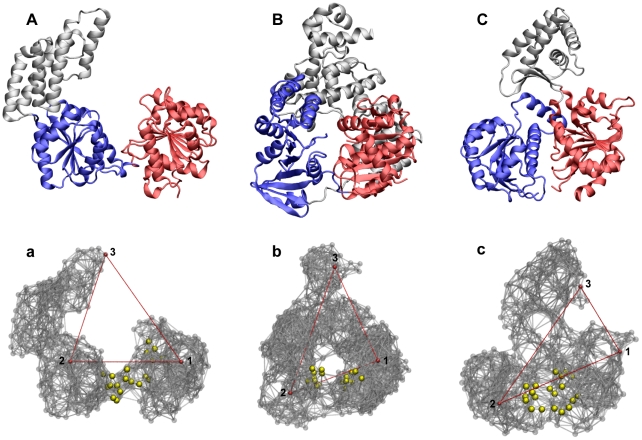
Superfamily 2 helicases. (A–C) Ribbon structures in the cartoon representation of Hef (A), Hel308 (B) and XPD helicase (C) and their respective elastic networks (a), (b) and (c). In the ribbon representation, the two RecA-like motor domains are colored red (domain1) and blue (domain 2). In the network representation, three labels 1,2 and 3 are indicated. The distances between them, indicated by red lines, are used for visualization of conformational relaxation. The residues belonging to the conserved ATP binding motifs are highlighted as yellow spheres.

Figure 1 shows ribbon structures of the three considered helicase proteins and their respective elastic networks. The equilibrium conformations are 1WP9 (Hef, chain A), 2P6U (Hel308) and 2VL7 (XPD). Two motor domains and an additional third domain are present in all these proteins. It should be noted that these three helicases have not yet been co-crystalized with an ATP-analog and the corresponding ligand-induced structures are not known. Nonetheless, conserved residue motifs, common for all members of superfamily 2 helicases and associated with ATP binding, have been identified [30]–[32]. In the studied helicases (and HCV helicase), they are located at the interface between the two motor domains, suggesting that ATP molecules bind in this region.

Within the elastic network approximation, amino acid residues are replaced by identical beads and effective interactions between them are taken into account through empirical harmonic potentials. Two beads are connected by a deformable link if the distance between them, deduced from the crystallographic data of the known equilibrium conformation, is within a prescribed interaction radius. Details of the network construction are given in the Analysis section. Only slow conformational motions, with the time scales of milliseconds or longer, will be considered here. In such processes, dissipative forces dominate conformational dynamics and inertial effects can be neglected. If hydrodynamical effects and thermal fluctuations are not taken into account, conformational motions inside a protein represent, therefore, relaxation processes of its elastic network towards the equilibrium configuration with the minimal elastic energy.

The dynamics of the three selected helicases has been probed by monitoring relaxation of their elastic networks after application of various initial deformations. The dynamics has been determined by numerically integrating the equations of motion, which yields the positions of the network beads at all time moments. To visualize conformational changes we have selected three network beads as labels, each belonging to a different domain, and tracked temporal evolution of the distances between them. In this manner, any conformational relaxation process could be characterized by a trajectory in the three-dimensional space of normalized distance changes between the labels; the origin of coordinates corresponds then to the equilibrium conformation.

Two different kinds of initial deformations were considered. The deformations of the first kind were obtained by perturbing the structure of the elastic network globally, i.e. by applying random forces globally distributed over all network beads. In the second case, perturbations were localized in the ATP binding pocket and initial deformations were generated by applying random forces only to those network beads that corresponded to the residues of the conserved ATP binding motifs. In both cases, deformed conformations of the network were obtained by integrating equations of motion in the presence of random forces for a fixed time period (see Analysis).

**Figure 2:**
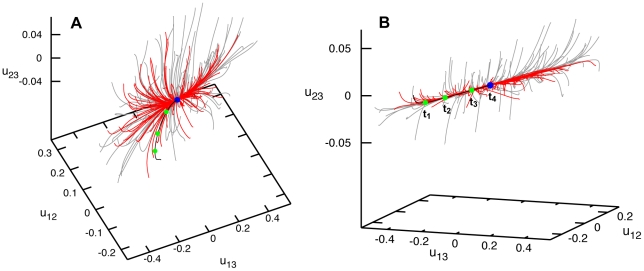
Relaxation dynamics of Hef helicase. Panels (A) and (B) show the relaxation pattern as a set of 100 trajectories in the space of normalized relative distance changes 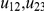 and 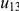 between the three labels. Each trajectory starts from a different initial deformation that has been generated by applying random static forces globally distributed to all network beads (grey trajectories) or restricted to the beads of the ATP binding site (red trajectories). The viewpoint in (B) is chosen in such a way that the planar confinement of relaxation trajectories is best seen. Note that the scale for the distance changes 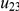 (the vertical axis) is much smaller than for the other axes.

Figure 2 shows the relaxation pattern for the elastic network of Hef helicase. Grey trajectories start from different initial deformations obtained by applying global perturbations. Remarkably, no meta-stable states could be found. Even though many initial states corresponded to highly deformed conformations, the elastic network of the protein could always return to its equilibrium shape. Examining further the relaxation pattern, we notice that, while distances between the labels 1 and 2 or 1 and 3 could vary up to 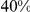, application of the same forces could change the distance between labels 2 and 3 by only a few percent. This suggests that the third domain in this protein is relatively rigidly attached to the second motor domain and they move essentially as a single object. Small initial changes 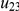 in the distance between domains 2 and 3 soon disappear and the relaxation trajectories become confined to a plane (see Fig. 2B), on which the subsequent slow relaxation takes place. We see that Hef helicase performs well-defined relaxation motions of the motor domain 1 with respect to domains 2 and 3, responding to random perturbation forces applied to all its residues.

Binding of an ATP ligand should produce local forces which are applied only to the residues in the binding pocket region. The exact details of such forces and of the induced responses in Hef helicase are not known. Nonetheless, we could generally probe the responses by applying various random static forces only to a subset of residues in the ATP binding pocket, similar to what we have previously done in Ref. [29] for the HCV helicase. Relaxation processes starting from the states induced by the application of such local forces are shown as red trajectories in Fig. 2. One can see that the protein responds to the local perturbations in the ATP binding region essentially in the same way as to the generic globally distributed perturbations (grey trajectories). Well-defined relative motions of the motor domain 1 are again observed.

**Figure 3:**
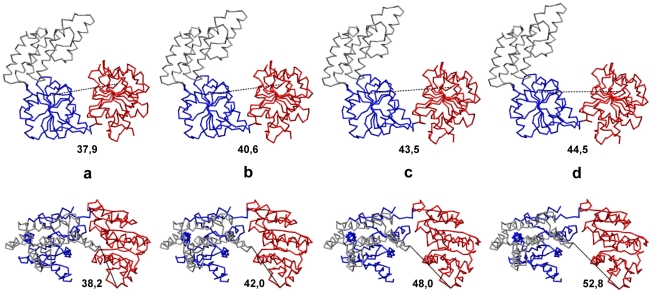
Conformational motions in Hef helicase. Consequent snapshots showing conformational changes in Hef helicase along the relaxation trajectory, highlighted in black in Fig. 2. Snapshots (a–d) correspond to the states at time moments 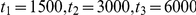 and 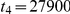 which are indicated by green dots on the trajectory. Both the front view (upper row) and the top view (bottom row) for all conformations are displayed. Absolute distances 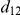 in Å between the two motor domains at the respective time moments are indicated under the snapshots in the upper row. In the bottom row, the corresponding distances 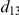 are given. Coloring of the domains is the same as in Fig. 1. The backbone 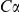 trace representation is employed.

To further characterize domain motions, the entire conformational relaxation process, corresponding to one particular trajectory (highlighted black in Fig. 2) can be watched in Video S1 in the backbone-trace representation. Several consequent snapshots from this video are shown in the front and top views in Fig. 3 (a–d). Large-amplitude hinge motions of the mobile motor domain 1 with respect to the other two domains, which are rigidly moving, are clearly seen. During conformational relaxation, the distance between the two motor domains increased from 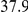 Å to 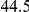 Å so that a large-amplitude conformational change was observed. The distance between the first motor domain and the domain 3 was also increasing from 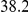 Å to 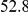 Å.

**Figure 4:**
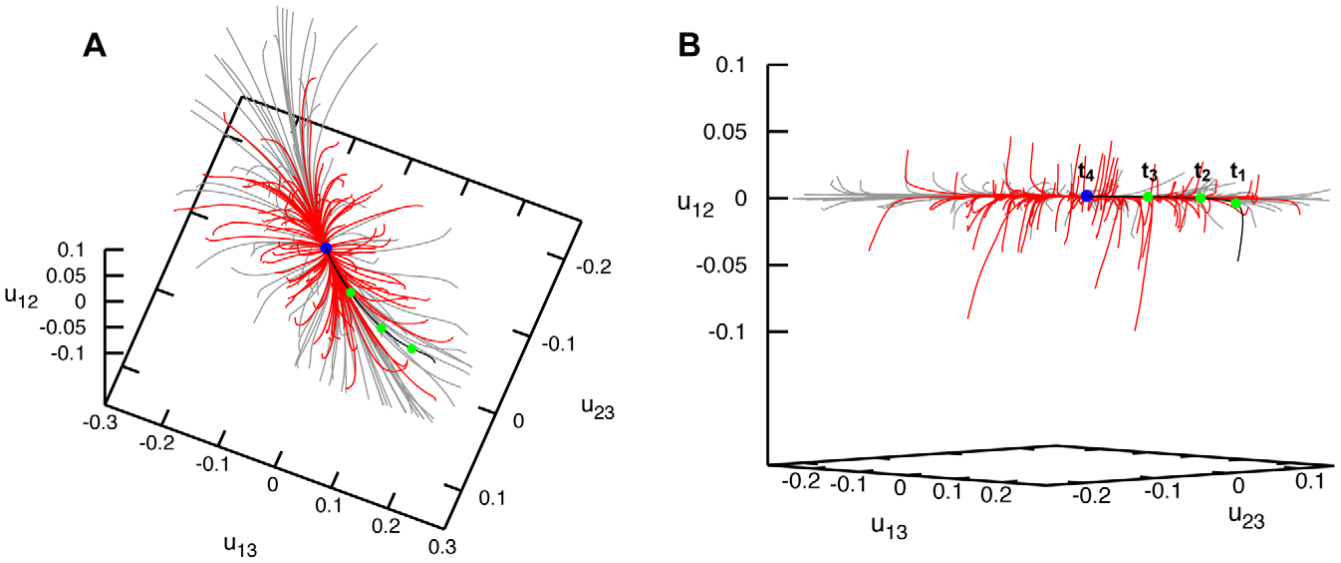
Relaxation dynamics of Hel308 helicase. Panels (A) and (B) show the relaxation pattern as a set of 100 trajectories in the space of normalized relative distance changes 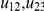 and 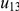 between the three labels. Each trajectory starts from a different initial deformation that has been generated by applying random static forces globally distributed to all network beads (grey trajectories) or restricted to the beads of the ATP binding site (red trajectories). The viewpoint in (B) is chosen in such a way that the confinement of relaxation trajectories is best seen.

By using the same methods, relaxation dynamics of the Hel308 elastic network has been investigated. Similar to Hef helicase, we found that the trajectories beginning from different initial deformations all returned to the equilibrium conformation with no meta-stable states present (Fig. 4). After short transients, trajectories converged to an attractive bundle along which the relaxation proceeded to the equilibrium conformation of the protein. This behavior was found for both sets of initial deformations, either obtained by applying random forces globally or with the random forces spatially confined to the ATP binding motifs.

In contrast to Hef helicase, the distance between the two motor domains, characterized by 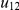, did not significantly change when perturbation forces were applied. On the other hand, large changes 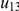 and 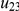 in the distance between the third domain and the two motor domains could be induced by the same random forces, with the relative deformations reaching about 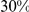. Thus, the generic soft conformational dynamics in Hel308 helicase corresponds to large-amplitude ordered motions of the third domain with respect to the two motor domains. It is remarkable that large-amplitude motions of this domain can be generated by mechanical perturbations that are spatially confined to the remote ATP binding pocket.

**Figure 5:**
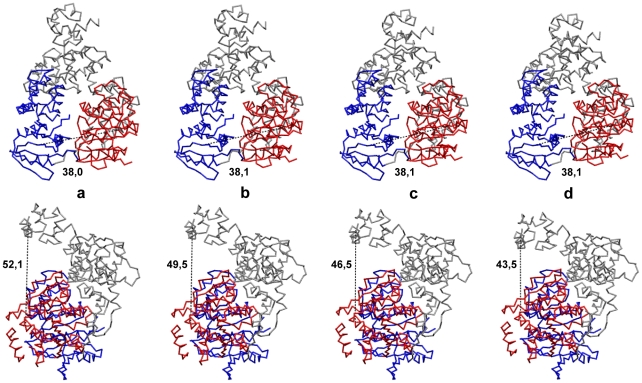
Conformational motions in Hel308 helicase. Consequent snapshots showing conformational changes in Hel308 helicase along the relaxation trajectory, highlighted in black in Fig. 4. Snapshots (a–d) correspond to the states at time moments 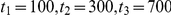 and 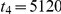 which are indicated by green dots on the trajectory. Both the front view (upper row) and the side view (bottom row) for all conformations are displayed. Absolute distances 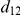 in Å between the two motor domains at the respective time moments are indicated under the snapshots in the upper row. In the bottom row, the corresponding distances 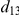 are given. Coloring of the domains is the same as in Fig. 1. The backbone 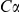 trace representations for different conformations is used.

To further illustrate the typical conformational relaxation process in Hel308 helicase, we show in Fig. 5 a series of four snapshots at time moments 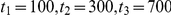 and 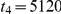 taken along the trajectory, which is outlined in black in Fig. 4. The relaxation can also be viewed as the Video S2. One can clearly see that the top part of the third domain is mobile and can move substantially with respect to the two motor domains (with the distance changes of about 9 Å).

**Figure 6:**
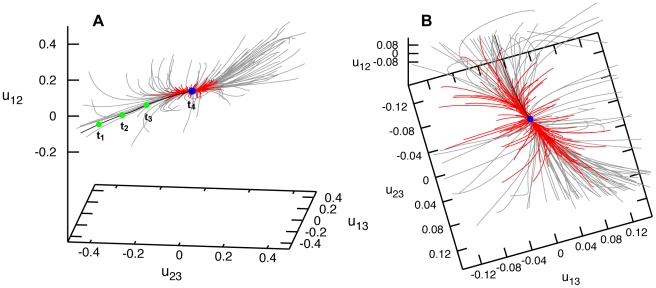
Relaxation dynamics of XPD helicase. Panels (A) and (B) show the relaxation pattern as a set of 100 trajectories in the space of normalized relative distance changes 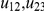 and 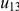 between the three labels. Each trajectory starts from a different initial deformation that has been generated by applying random static forces globally distributed to all network beads (grey trajectories) or restricted to the beads of the ATP binding site (red trajectories). In panel (B), a different viewpoint is chosen and the scales are smaller.

Finally, we present the results for XPD helicase, the last protein investigated. Relaxation processes starting from initial deformations generated by applying random forces to all network beads are shown as grey trajectories in Fig. 6. The initial induced deformations were strong, as evidenced by large relative distance changes between the labels (up to 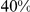). It can be moreover noted that, in this helicase, the perturbations induced pronounced rearrangements of all three domains. Nonetheless, the trajectories returned to the equilibrium conformation and meta-stable states were not found.

Relaxation trajectories starting from the second set of deformations, obtained by applying random forces only to the residues in the ATP binding pocket, are shown as red curves in Fig. 6. As we see, deformations induced by such spatially localized forces were significantly smaller, with the relative distance changes between the labels not exceeding 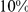. Even when stronger forces were applied, much larger deformations could not be found in our simulations (not shown in the figure). Thus, large conformational changes cannot be generated by mechanically perturbing residues of the ATP binding pocket only. This finding is in contrast to what we have observed for the other two helicase proteins (and also for the previously studied HCV helicase [29]).

**Figure 7:**
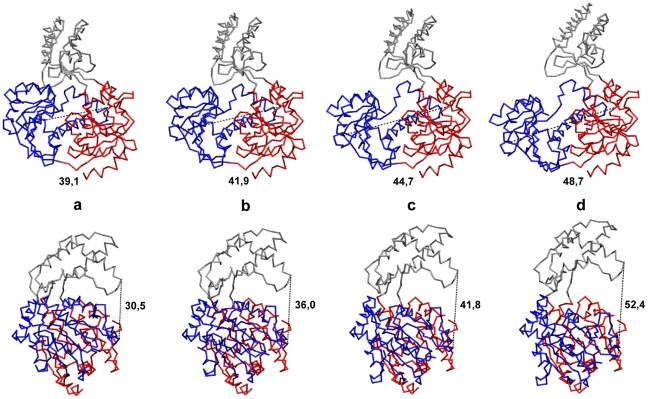
Conformational motions in XPD helicase. Consequent snapshots showing conformational changes in Hel308 helicase along the relaxation trajectory, highlighted in black in Fig. 6. Snapshots (a–d) correspond to the states at time moments 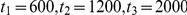 and 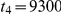 indicated by green dots on the trajectory. Both the front view (upper row) and the side view (bottom row) for all conformations are displayed. Absolute distances 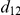 in Å between the two motor domains at the respective time moments are indicated under the snapshots in the upper row. In the bottom row, the corresponding distances 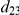 are given. Coloring of the domains is the same as in Fig. 1. The backbone 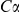 trace representation is used.

Large-amplitude domain motions accompanying one particular relaxation process (corresponding to the black trajectory in Fig. 6) are shown in Video S3 in the backbone-trace representation. Snapshots from this video are displayed in Fig. 7. During conformational relaxation large changes in the relative positions of motor domains can be seen (around 10 Å) and absolute changes in the distance between the third domain and the motor domain 2 are even larger.

## Discussion

The objective of this paper was to investigate conformational dynamics of proteins in the largest helicase superfamily in the framework of a coarse-grained mechanical description. Viewed as elastic objects, proteins were modeled as networks of identical beads connected by deformable springs. Three representative helicases of the superfamily 2 were chosen by us, i.e. the Hef, the Hel308 and the XPD helicases. To study conformational motions of the proteins, we have probed the dynamics of their corresponding elastic networks in response to various mechanical perturbations.

The common observed property of all studied helicases is that they respond in a well-defined way to mechanical perturbations and their responses consist of large-amplitude relative changes in the positions of protein domains. Though the distances between the domains may change up to 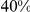, such conformational changes can still take place without protein unfolding, with the elongations of elastic links remaining relatively small. The relaxation starting from deformed initial conformations involves ordered motions of major protein domains. Metastable states could not be found and the molecules always returned to the original equilibrium conformation. Previously, similar behavior has been observed for other motor proteins [27], [28] and it has also been found by us for the molecular motor HCV helicase [29]. The ability of motor proteins to perform ordered conformational motions triggered by ATP binding and hydrolysis is a fundamental property of molecular machines.

On the other hand, we have also seen important differences in the conformational dynamics of the three studied proteins. These differences can be important for the biological operation of the helicases, i.e. for the processing of nucleic acid substrates. Below, the functional aspects are discussed separately for each of the studied molecules.

### Hef helicase

**Figure 8:**
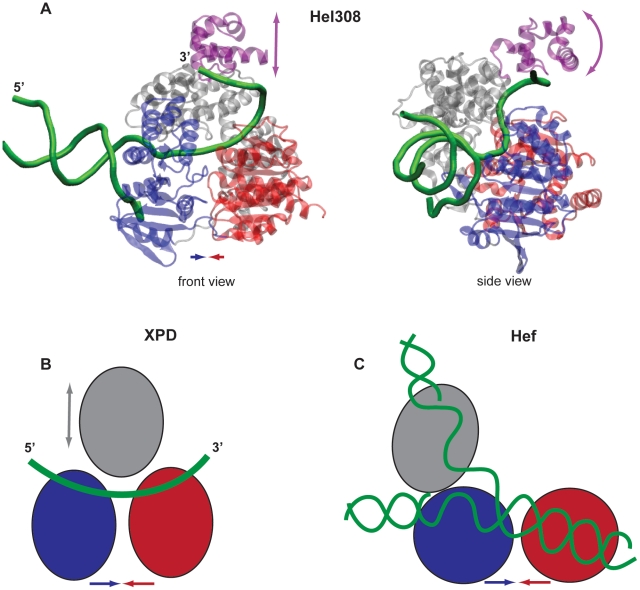
Helicase function on DNA. (A) Structure of Hel308 helicase with partially unwound DNA bound to it (2P6R) in the front and side views. The DNA strands are shown as green; the direction of translocation along the unwound single strand is 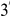 to 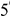. The coloring of the domains is the same as in the other figures, but the top part of domain 3, referred to as the arch subdomain, is colored in purple here. (B) Schematic drawing of XPD helicase with the single DNA translocation strand positioned according to Ref. [32]. In this protein, the direction of translocation is 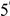 to 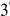. (C) Possible orientation of the branched duplex DNA in Hef helicase as proposed in Ref. [30] (schematic drawing). In all panels, arrows indicate possible domain motions.

The structure of this protein complexed with DNA is still not available. Nonetheless, conserved motifs crucial for DNA binding have been identified in the two motor domains [30]. Analysis of the electrostatic surface potential and mutational studies of the protein have revealed that the third domain can recognize and bind specific (e.g. fork-structured) DNA. According to the model in Ref. [30], the two motor domains can bind double- or single-stranded DNA (see Fig. 8). Our study shows that in Hef helicase a mobile motor domain is able to perform large-amplitude hinge motions with respect to the other motor domain and the third domain, which are rigidly connected. This observation is in accordance with what has been previously proposed based on the crystallographic studies of this protein [30]. The hinge motion brings together or spatially separates, respectively, the residues of the conserved motifs which are located on the two motor domains. These motifs are involved in binding and hydrolysis of ATP and thus it is likely that such conformational motions are functional and essential for the motor operation. Our investigations suggest, that, in Hef helicase, DNA is actively transported by the two motor domains and is further processed by the third domain. This behavior closely resembles that of the HCV helicase, where large relative motions of the motor domains are enabling hydrolysis of ATP and drive progressive translocation along the nucleic acid [29]. Remarkably, both in Hef and HCV helicase, the motions of the motor domain can be induced by the forces applied only in the ATP binding region.

### Hel308 helicase

The structure of this protein with partially unwound DNA (2P6R) reveals that the duplex DNA is bound to motor domain 2 and becomes separated by this domain [31]. The unwound 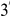 tail winds through the entire protein having contacts with all its domains. The backbone of the DNA strand goes across both motor domains, establishing contacts to the residues of the conserved nucleic acid binding motifs. Then it meanders towards the arch subdomain, binding also there [31] (see Fig. 8). The proposed operation mechanism of this helicase [31] consists of the processive helicase translocation in the 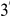-to-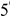 direction accompanied by the ATP-dependent ratchet-like transport of the DNA strand, involving relative motions of the motor domains (inchworming). Mutational studies have revealed that the arch subdomain is of special importance for the helicase function. It has been found that the absence of this subdomain results in a significantly higher helicase processivity, as compared to the wild-type protein, and, therefore an autoinihibitory or molecular brake role has been assigned to the arch subdomain [33]. Thus, it has been previously suggested that its function can be to limit and control the helicase activity. While our present investigations have shown that, in contrast to Hef helicase, the motor domains are already close one to another in the native conformation of this protein and their relative motions are much less pronounced, the position of the DNA strand within the Hel308-DNA complex still suggests that such motions may be used for the ATP-dependent translocation. We have found, additionally, that the top part of the third domain, representing the arch subdomain [31], could perform large-amplitude hinge motions with respect to the two motor domains. Such motions could be induced both by globally distributed perturbations and by the local forces applied only in the ATP binding pocket. As we have seen, conformational changes in the arch subdomain could be produced by the perturbations acting in the ATP binding pocket, indicating correlations between the ATP binding or hydrolysis and the regulation of helicase activity and thus the presence of longe-range internal communication in this protein. Such possible correlations have previously been also discussed based on the biochemical analysis [34]. Our simulation results suggest that the arch subdomain does not merely function as a passive brake, but has an active functional role. It can mechanically regulate the grip on the 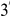 DNA tail in an ATP-dependent fashion. Thereby, the arch subdomain may actually operate as a clamp to control the activity on the DNA substrate.

### XPD helicase

Structural data for the XPD helicase-nucleic acid complex is not yet available. Nonetheless, the conserved residue motifs, known to interact with the nucleic acid substrate in other helicases, have been identified in this protein [32]. They are located on the surface of the two motor domains. Using this data, the single-strand of DNA can be positioned like in the well-resolved Hel308-DNA complex [32] (see Fig. 8). We have found that, in XPD helicase, significant changes in relative positions of all three domains accompany conformational dynamics. However, in contrast to the two other helicases (and HCV helicase), they could not be generated by mechanically perturbing residues only in the ATP binding site. Thus, additional mechanical stimuli, provided, e.g., by the interactions with the nucleic acid substrate, may be needed in this protein to yield significant motions of the motor domains. For a different helicase in the same superfamily 2, it could indeed have been shown that binding of the nucleic acid to the apo structure may induce large repositioning of the motor domains, thus affecting the binding affinity of ATP molecules to the protein and controlling the ATP-dependent helicase activity [11]. We cannot however also exclude a possibility that the conserved residue motifs, which have so far been identified for XPD helicase, do not actually account for all residues constituting the ATP binding pocket. It may therefore turn out that large-scale conformational changes may still be induced by local perturbations in this pocket, but the forces should then be applied to some other residues. Indeed, in the crystal structure used by us, residues of the conserved ATP binding motif V could not be determined and were therefore missing in our simulations.

Generally, one can expect that the dynamical behavior of helicases, regarded as a result of biological evolution and selection, should be adapted to particular molecular processes in which they are involved and thus to the specific forms of the nucleic acid substrate. In viruses, where helicases are functioning in the molecular replication machinery together with polymerase proteins, they need to translocate over large distances along nucleic acid strands and separate their duplex structure, in order to ensure efficient multiplication of the viral genome. The situation is different when branched forms of nucleic acids should be separated during transcription processes or intermediate bubbles in regular duplex structures should be generated in the processes of DNA repair. In this case, helicases need to catalyze only local unwinding of short duplexes and aspects of efficient translocation might be less important. Instead, the execution of specific functions, such as nucleic acid recognition and fine-regulated processing of nucleic acids, can play then a dominant role in conformational dynamics.

Two possible operation mechanisms have previously been discussed for superfamily 2 helicases [9], [35]. According to the active mechanism, a helicase can progressively move itself along the nucleic acid substrate, thereby destabilizing its duplex structure and eventually separating the strands. The passive mechanism assumes instead that, once a base pair becomes broken due to thermal melting, the helicase passively moves along the strand and locally locks the destabilized region. In our recent study of HCV helicase [29], we could reproduce the entire operation cycles of active nucleic acid unwinding, with the motor domains acting as a translocation machine. In that study, interactions with the DNA strands have been explicitly included into the considered dynamical model. For the three presently chosen helicases, we could not yet consider entire operation cycles, including interactions with nucleic acids and unwinding processes. We can, however, note that, when large relative motions of motor domains are observed, this may indicate the active mode of operation including stepwise translocation along the nucleic acid strand, though further investigations are needed to clarify the situation.

Any coarse-grained models, including EN descriptions, are intrinsically limited in their explanatory power and cannot fully describe molecular operation mechanisms of motor proteins. While chemical details are not resolved in elastic network models, it is, however, remarkable that such purely mechanical descriptions are still able to capture essential aspects of functional dynamics in protein motors. Apparently, the operation of molecular motors is so robust that, in a rough approximation, it is not sensitive to fine chemical details. In the future it can be however interesting to develop and apply hybrid descriptions, combining coarse-grained elastic network models with more realistic MD simulations.

## Analysis

Within the elastic network description, a protein is viewed as an elastic object representing a network of 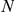 identical beads connected by deformable elastic links. Each bead 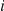 corresponds to a 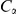-atom of an amino acid residue. Equilibrium positions of the beads are specified by vectors 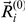. They are taken from the RCSB Protein Data Bank for the respective protein. Two beads 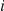 and 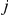 are connected by an elastic spring if at equilibrium the distance 
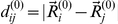
 between them is shorter than some chosen interaction radius 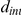. The architecture of the protein network is described by the connectivity matrix 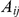 with the elements 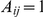, if 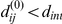, and 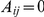, else. In our simulations the value of 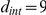 Åhas been used. The choice of the interaction radius in the elastic-network models is to some extend arbitrary (cf. [36], [37]). It cannot be chosen to be too small, because then the network tends to include weakly connected residue groups or even disconnected components. The interaction radius cannot be also too large, because then many cross-links between the domains become present, preventing large-scale domain motions. Still, this parameter can usually be chosen in different ways. In the literature, the values varying from 8 Å to 10 Å can be found. Before selecting a particular interactions radius in our study, we have performed a series of preliminary simulations with the elastic networks constructed by using its different values. The interaction radius of 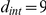 Å lies in the middle of the interval of physically possible parameter values. We have checked in several simulations that the results do not considerably change if the interaction radius is slightly increased of decreased.

In a deformed state, the positions of the beads 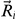 are changed and the lengths 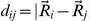 of elastic springs are generally different from their natural lengths 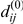. Note that the connectivity matrix 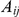 is based on the native conformation of the protein and it is not allowed to change when the network gets deformed. Therefore, only elastic conformational motions, not accompanied by (partial) unfolding or refolding can be considered in this approach.

The elastic energy of a network is
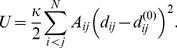
(1)All springs have the same stiffness constant 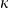. By construction, the energy minimum 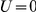 corresponds to the experimentally known native conformation.

Hydrodynamical effects and thermal fluctuations are neglected in this simplest model. Moreover, only slow processes are considered, where the inertial forces can be neglected and the overdamped limit applies. Under these approximations, the dynamics of the network obeys a set of 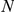 differential equations for the motions of the beads. The velocity of a bead (i.e. the time-derivative of its position vector) is proportional to the total forces applied to it. Thus, we have
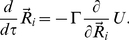
(2)Here, 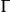 is the mobility of a bead, assumed to be the same for all beads. By using the expression (1) for the elastic energy 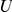, these equations are written explicitly as
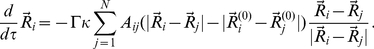
(3)The elastic forces depend linearly on the elongations of springs connecting the beads. Nonetheless, the dynamics of the network is still nonlinear, since distances are nonlinear functions of spatial coordinates, i.e. 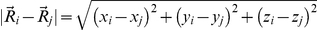. By introducing the dimensionless rescaled time 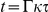, the spring constant 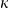 and the bead mobility 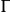 are eliminated from the evolution equations.

To generate initial deformations, external forces 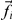 were applied to the beads. Thus, the dynamical equations used in the simulations were
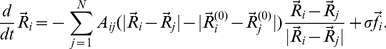
(4)


For convenience, we have introduced here a switching parameter 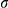. When 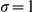, external forces are switched on, while for 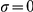 they are switched out. The dynamical equations (4) can be numerically integrated to obtain the positions of all network beads at various moments.

Since our study aims to investigate dynamical responses of helicases to deformations of their elastic networks, we needed to prepare various deformed states. The first set of initial deformations was generated by applying random static forces which were globally distributed over all network beads. Specifically, we generated forces 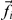 acting on beads 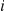, by choosing the components 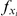, 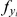 and 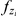 as random numbers from the interval between 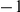 and 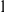 and, after that, rescaling the forces in such a way that the normalization condition 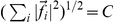 was always satisfied. To obtain the coordinates of beads in the initial deformed state, equations (4) were numerically integrated in the presence of forces (i.e. with 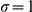) for a fixed time 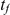. This procedure was repeated to prepare a set of 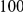 initial network deformations, each arising from a different random configuration of forces. For each prepared initial deformation we have checked that the springs were not excessively stretched, i.e. that the plastic deformations were excluded. Namely, we have required that elongations of the springs do not exceed 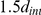 in the initial deformed states. The numerical values used in the simulations were 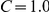 Å and 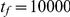 for Hef, 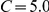 Å and 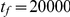 for Hel308 and 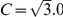 Å and 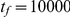 for XPD.

In motor proteins, ligand-induced conformational motions are of particular interest. Since none of the chosen helicases have been so far co-crystallized with an ATP-analog, details of their interactions with ATP molecules are not known. Nonetheless, residue motifs that are conserved have been identified in all three helicases [30]–[32]. Conserved motifs involved in binding of ATP molecules are located at the interface between the motor domains, suggesting that ATP molecules bind there. Assuming that interactions between ATP and the helicases produce local forces in this region, we have probed helicase dynamics in response to mechanical perturbations confined to the binding pocket. To do this, we have generated another set of initial deformations in a similar way as described above, but with the difference that the random static forces were applied only to the network beads that corresponded to the residues of the conserved ATP binding motifs in each of the helicases.

The following residue motifs were used in our simulations. For the Hef helicase: 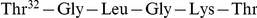 (motif I), 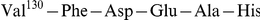 (motif II) and 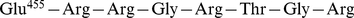 (motif VI). For the Hel308 helicase: 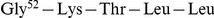 (motif I), 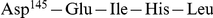 (motif II) and 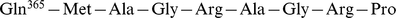 (motif VI). For the XPD helicase: 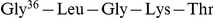 (motif I), 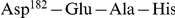 (motif II), 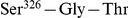 (motif III) and 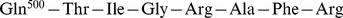 (motif VI). In these simulations, the values 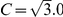 Å for Hef, 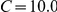 Å for Hel308 and 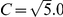 Å for XPD have been used. The values for 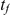 were the same as before.

Starting from various deformed conformations, we switched off the forces in the second part of our simulations (setting the parameter 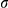 to zero) and integrated equations (4) to study relaxation dynamics starting from the chosen initial states.

To visualize conformational changes, we have tracked time evolution of the distances between three selected labels. Relaxation processes were therefore represented by trajectories in the three-dimensional space of these coordinates. For presentation of the relaxation patterns, normalized relative distance changes between the labels 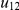, 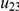 and 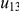, with, e.g., 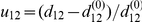, were chosen. Labels 1 and 2 were chosen to lie in the motor domains 1 and 2, whereas label 3 belonged to domain 3. For Hef helicase the labels 1,2 and 3 were 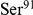, 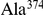 and 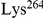. For Hel308 helicase we have taken 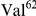, 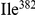 and 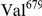. For XPD helicase we have taken 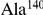, 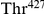 and 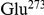.

Figures and movies that visualize helicase conformations and structural changes have been prepared using Visual Molecular Dynamics (VMD) [38].

## Supporting Information

Video S1Domain motions in Hef helicase accompanying a conformational relaxation process that corresponds to one particular trajectory (highlighted black in Fig. 2). The Video starts at time moment 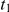 and the protein is displayed in the backbone-trace representation. The front view (left side) and top view (right side) is provided.(MOV)

Video S2Domain motions in Hel308 helicase accompanying a conformational relaxation process that corresponds to one particular trajectory (highlighted black in Fig. 4). The Video starts at time moment 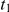 and the protein is displayed in the backbone-trace representation. The front view (left side) and side view (right side) is provided.(MOV)

Video S3Domain motions in XPD helicase accompanying a conformational relaxation process that corresponds to one particular trajectory (highlighted black in Fig. 6). The Video starts at time moment 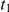 and the protein is displayed in the backbone-trace representation. The front view (left side) and side view (right side) is provided.(MOV)
